# Intravitreal Expansile Gas and Bevacizumab Injection for Submacular Hemorrhage Due to Neovascular Age-related Macular Degeneration

**Published:** 2010-07

**Authors:** Ramin Nourinia, Mohammad Hossein Jabbarpour Bonyadi, Hamid Ahmadieh

**Affiliations:** 1 Urmia University of Medical Sciences, Urmia, Iran; 2 Ophthalmic Research Center, Labbafinejad Medical Center, Shahid Beheshti University of Medical Sciences, Tehran, Iran

**Keywords:** Bevacizumab, Submacular Hemorrhage, Macular Degeneration

## Abstract

**Purpose:**

To evaluate the results of intravitreal expansile gas injection, with or without recombinant tissue plasminogen activator (rtPA), followed by intravitreal bevacizumab injection for treatment of submacular hemorrhage (SMH) secondary to neovascular age-related macular degeneration (AMD).

**Methods:**

In this interventional case series, 5 eyes of 5 patients with SMH secondary to choroidal neovascularization (CNV) due to neovascular AMD were treated with 0.3 cc intravitreal SF6 (and 50 μg of rtPA in two eyes), followed by face-down positioning; 24 hours later, 1.25 mg of bevacizumab was injected intravitreally. Main outcome measures included displacement of SMH and best corrected visual acuity (BCVA).

**Results:**

Mean patient age was 75.6±9.2 (range, 60–83) years, mean duration of symptoms was 6.4±3.2 (range, 3–10) days, and mean number of bevacizumab injections was 1.8 (range, 1–3). Mean preoperative BCVA was 1.28±0.27 logMAR which improved significantly to 0.57±0.33 logMAR at 12 months (P=0.042). SMH displacement occurred in all eyes, and visual acuity improved and remained stable during the follow-up period of 12 months.

**Conclusion:**

Intravitreal expansile gas injection, with or without rtPA, followed by intravitreal bevacizumab injection, seems to be an effective modality for SMH displacement and treatment of the underlying CNV in neovascular AMD.

## INTRODUCTION

Thick submacular hemorrhage (SMH), particularly in patients with neovascular age-related macular degeneration (AMD), is generally associated with poor visual prognosis. Retrospective natural history studies have demonstrated that final visual acuity (VA) in AMD patients with thick blood beneath the fovea is rarely better than 20/200.^1^–^4^ Several mechanisms, including shearing of photoreceptors by fibrin clots, physical separation of the photoreceptors from the retinal pigment epithelium and iron toxicity have been suggested as explanations for retinal damage caused by thick subretinal blood.^5^–^7^ Natural history and experimental data have prompted the search for a safe and effective method of removing the blood from beneath the macula to hasten visual recovery and prevent irreversible damage to the outer retina.^7^–^12^ There is no standard treatment for acute SMH; the effectiveness of various treatment approaches is difficult to evaluate and final VA depends to a great extent on the cause and extent of macular pathology. Advances in vitreoretinal surgery have made the removal of submacular blood possible, but surgical intervention is associated with various complications.

Several reports have demonstrated the ability of an intravitreal gas bubble, with^13^–^16^ or without^17^,^18^ intravitreal recombinant plasminogen activator (rtPA), to displace thick blood from beneath the fovea in most eyes. Visual improvement is often limited by the initial location and progression of the choroidal neovascularization (CNV), regardless of the technique used to treat SMH. Therefore, early diagnosis and treatment of the underlying cause of SMH may be the key to improving visual outcomes. Bevacizumab, a pan-VEGF inhibitor, has been demonstrated to possess the ability to reduce vascular leakage and inhibit growth of the CNV and has been widely adopted for treatment of neovascular AMD.^19^ We hypothesized that intravitreal application of gas, with or without rtPA, supplemented by bevacizumab, would be beneficial in eyes with SMH due to neovascular AMD.

## METHODS

This non-randomized interventional case series includes 5 eyes with SMH due to neovascular AMD that received intravitreal injection of expansile gas with or without rtPA followed by bevacizumab. Pre- and postoperative ophthalmologic examinations included determination of best corrected VA using a Snellen chart, slitlamp biomicroscopy, and indirect ophthalmoscopy. All patients were examined preoperatively and were followed for 12 months after the procedure. Color fundus photography, fluorescein angiography (FA), and optical coherence tomography (OCT) were performed. All SMHs were of sufficient thickness to induce significant elevation of the entire neurosensory retina, completely obscuring the underlying choroidal vascular pattern on biomicroscopic examination. Each patient gave informed consent to this novel treatment approach after the nature of the disease, its natural course and alternative treatment modalities had been explained.

The procedure was performed under sterile conditions in the operating room. Intravitreal injection was performed 3.75 mm and 3 mm behind the limbus in phakic and pseudophakic eyes, respectively, using a #30 gauge needle. First, commercial rtPA, a lyophilized material, was diluted with balanced salt solution to a concentration of 1 mg ml in polypropylene syringes. Then, 0.05 ml of the preparation, equal to 50 μg rtPA, was injected intravitreally. Subsequently, 0.3 ml of pure sulfur hexafluoride (SF6) gas was injected into the vitreous cavity. All patients were instructed to maintain face-down position until the next day. Twenty-four hours later, intravitreal injection of 1.25 mg/0.05 ml bevacizumab was performed in a similar fashion and under the same setting as described above.

Patients received reinjection(s) of bevacizumab when recurrent CNV was noticed. Recurrence was defined as a decrease in VA associated with new foci of subretinal hemorrhage or intraretinal fluid detected by OCT, and leakage noted by FA.

## RESULTS

Patient characteristics are shown in Table 1. Mean age was 75.6±9.18 years (range, 60–83 years) and mean duration of symptoms was 6.4±3.2 days (range, 3–10 days). Successful inferior displacement of the SMH was achieved in all (5/5) eyes. The mean number of bevacizumab injections was 1.8 (range, 1–3). Mean preoperative VA was 1.28±0.27 logMAR, which improved significantly to 0.57±0.33 logMAR at 12 months (P =0.042). No complications related to intravitreal injection of gas, rtPA, or bevacizumab occurred during the follow-up period.

Case 1 is presented below as an example of the subjects.

### Case 1

**Figure 1:**
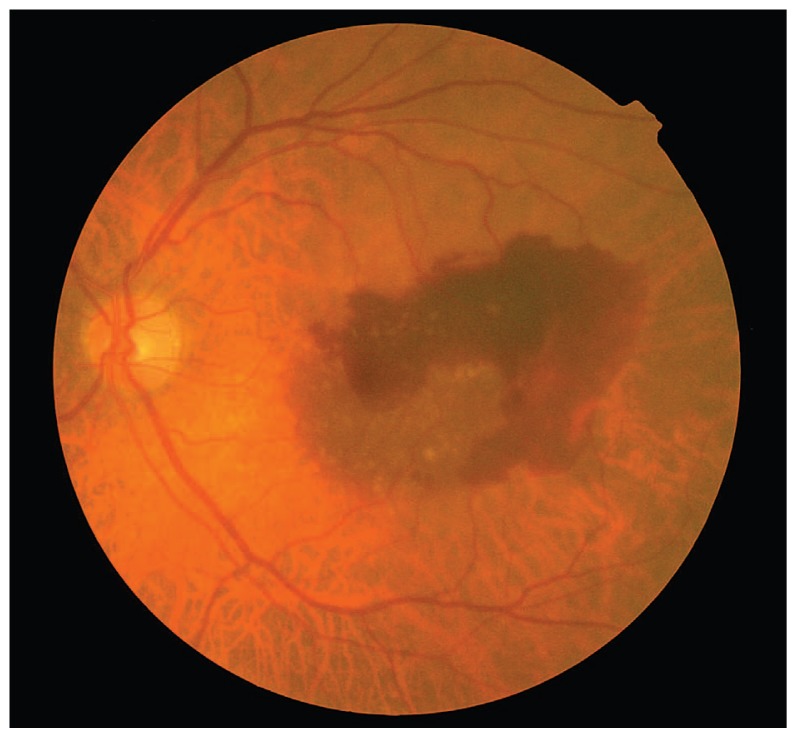
Color photograph of the left eye of case #1 before treatment reveals submacular hemorrhage.

**Figure 2:**
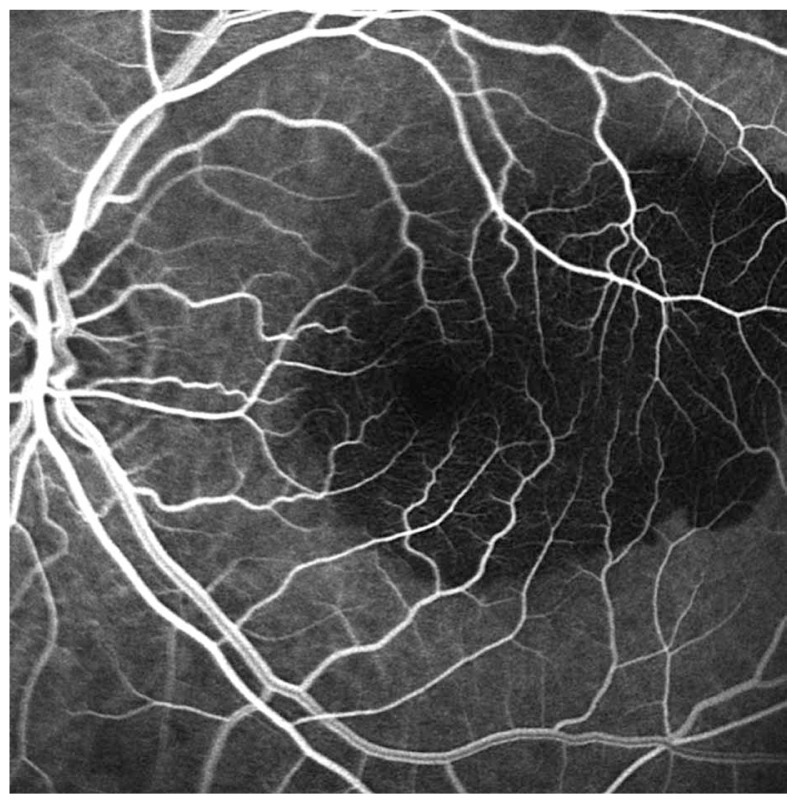
Fluorescein angiogram of the same eye as in figure 1 before treatment demonstrates fluorescein blockage due to submacular hemorrhage.

**Figure 3:**
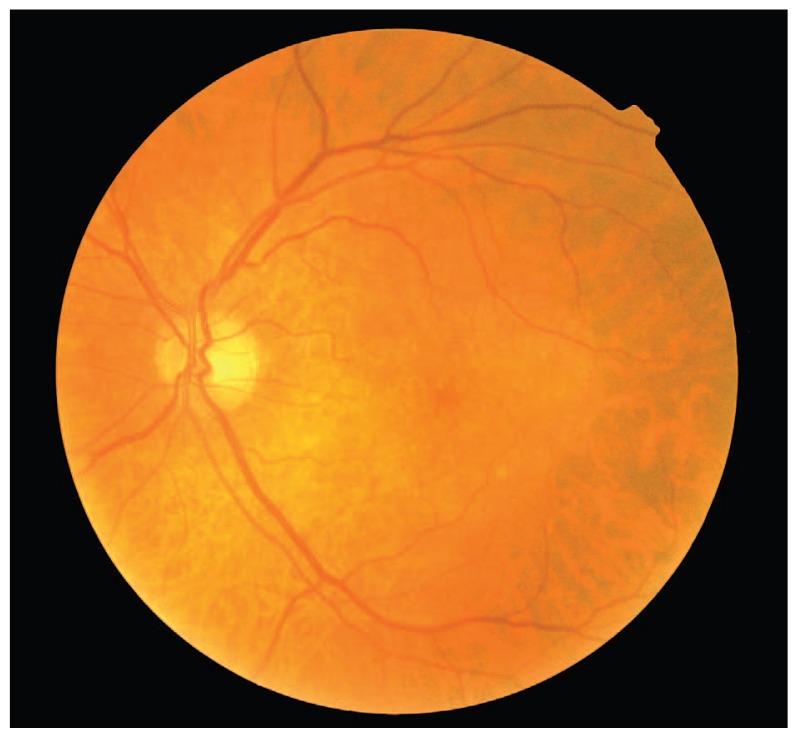
Color photograph of the left eye of case #1 demonstrates resolved submacular hemorrhage without scar formation 12 months after treatment.

**Figure 4:**
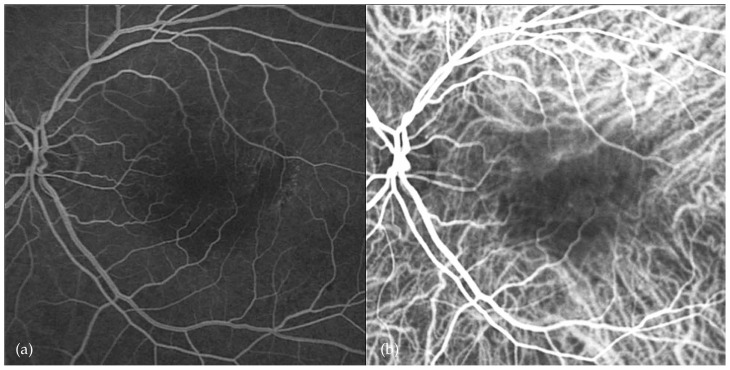
Fluorescein **(a)** and indocyanine green **(b)** angiography shows an inactive choroidal neovascular lesion 12 months after treatment in the left eye of case #1.

**Figure 5:**
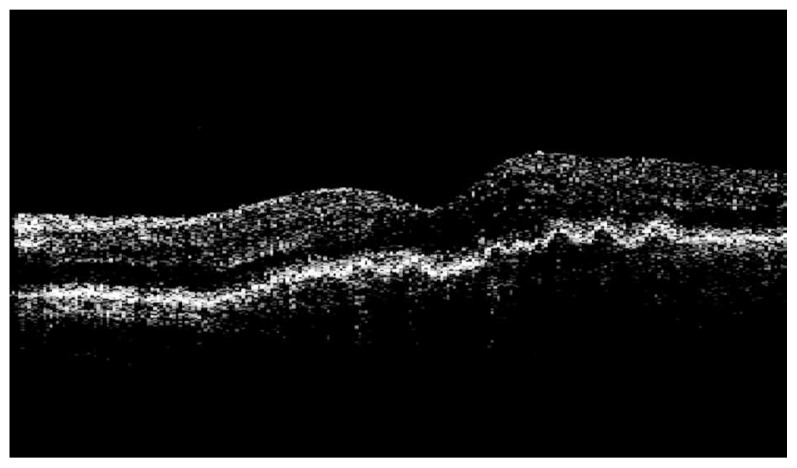
Post-treatment OCT image of the macula in the left eye of case #1 reveals no subretinal or intraretinal fluid 12 months after intervention.

An 80-year-old male patient had been under observation for dry-type AMD in his left eye. There was history of severe vision loss in the right eye due to a disciform scar secondary to AMD since 1995. VA in his left eye had been 20/25 with confluent soft drusen in the macula. In September 2006, he noticed sudden visual loss to 20/200. Ophthalmoscopy disclosed subretinal hemorrhage in the macular region (Fig. 1). Fluorescein angiography showed fluorescein blockage due to submacular hemorrhage (Fig. 2). An intravitreal injection of 50 μg rtPA and 0.3 ml SF6 was performed promptly, followed by prone positioning for 24 hours. Displacement of subretinal hemorrhage was apparent the next day and intravitreal injection of 1.25 mg bevacizumab was performed. Due to persistent leakage on FA, a second injection of the same dose of bevacizumab was performed one month later. Follow-up examinations were performed at six-week intervals using OCT. The patient was followed for one year; at final examination the CNV regressed and best corrected VA reached 20/30 (Figures 3, 4, 5).

## DISCUSSION

In this small case series, intravitreal injection of expansile gas with or without rtPA injection and followed by bevacizumab, proved successful in anatomical displacement of SMH and was associated with visual improvement. Mean preoperative VA was 1.28±0.27 logMAR which improved significantly to 0.57±0.33 logMAR at 12 months.

The visual outcome of AMD patients with submacular hemorrhage is typically poor, particularly in eyes with thick blood clots or those involving large areas of the macula and in the presence of a choroidal neovascular membrane. The size of the CNV and the amount of remaining SMH affect the volume of the disciform scar. Four reports have been published describing the natural course of submacular hemorrhage.^1^–^4^ Bennet and colleagues^1^ reported 29 patients with submacular hemorrhage; mean initial VA was 20/1300 and mean final VA was 20/1700. During follow-up, VA deteriorated or remained at the same level in all eyes. Avery and coworkers^3^ retrospectively reviewed a larger number of patients who had submacular hemorrhage secondary to AMD. Follow-up analysis was carried out at 12 and 36 months. Forty-one eyes were initially examined; after 12 months, 23 eyes were examined, and at 36 months only 15 eyes were evaluated. Mean initial VA was 20/200. By 36 months, 44% lost 6 or more lines of VA, 22% lost 2 to 5 lines, 12% retained vision within 1 line, and 21% showed spontaneous improvement of 2 or more lines. Of the group that improved, the most significant improvement occurred during the first 12 months, but even in this group vision deteriorated with longer follow-up. The largest series to date reviewed 60 eyes with submacular hemorrhage and AMD.^4^ As in prior studies, VA and the size and elevation of the hemorrhage were examined. Mean initial VA was 20/240 and mean VA of all 60 eyes after 24 months was 20/1250. Vision deteriorated in 80% of cases. Time for disappearance of the hemorrhage ranged from 2 to 18 months, with an average of 6 months.^4^ These studies support the clinical impression that the natural course of submacular hemorrhage is poor.

The Submacular Surgery Trial investigated AMD patients with SMH and reported decrease of two or more lines in VA in 64% of eyes with untreated SMH after 36 months of follow-up, as compared to 51% of eyes undergoing mechanical removal of the underlying CNV and SMH by pars plana vitrectomy.^20^ No significant difference in VA was observed between the treated and non-treated groups.

Heriot^21^ first described management of submacular hemorrhage with intravitreal rtPA injection and pneumatic displacement of blood from the fovea. His initial experience suggested a high anatomic success rate with few complications. Other reports subsequently confirmed the capability of an intravitreal gas bubble, with or without intravitreal rtPA, to displace thick blood from beneath the fovea in most eyes. This office-based procedure was simpler, less costly and less traumatic to photoreceptors and the retinal pigment epithelium as compared to surgical evacuation of subretinal blood.^22^ In a large series involving 52 cases, Chen et al^23^ demonstrated improvement of two Snellen lines or more, 3 months after rtPA and expansile gas injection. In another study, Regillo^24^ demonstrated that submacular blood was completely displaced from the fovea in 10 (71%) of 14 eyes and partially displaced in three (21%) eyes after intravitreal injection of rtPA and expansile gas. In one patient, no displacement occurred. Early postoperative VA (within 2 months) improved by two or more lines in eight eyes (57.1%). However, with a longer mean follow-up of 7.7 months, only 2 of the 14 eyes (14.2%) maintained 2 or more lines of improvement. No clinical evidence of retinal toxicity was seen.

Previously, additional intervention with photodynamic therapy (PDT) was employed if the SMH was shifted successfully from the fovea and the underlying CNV became visible. Bakri et al^25^ reported patients with subretinal hemorrhage who were noted to have foveal involvement due to neovascular AMD. Mean initial visual acuity was 20/372 which reached 20/227 twelve months after PDT treatment. The difference in VA was not statistically significant.

Antiangiogenic agents have opened a new approach toward effective and early treatment of choroidal neovascularization. The consecutive application of antiangiogenic agents treats the CNV which is the underlying cause of SMH. Stifter et al^26^ evaluated the effectiveness and safety of intravitreal bevacizumab alone, without displacement of the SMH, for large submacular hemorrhages in neovascular AMD, and reported visual stabilization in all eyes. Visual improvement of one line or more, and three lines or more was achieved in 38% and 9.5% of eyes, respectively.

Meyer et al^27^ reported that injection of intravitreal rtPA, expansile gas, and bevacizumab was successful in achieving anatomical displacement of the SMH. Mean VA improvement was 2.1 lines and 3.7 ETDRS chart lines 1 and 3 months after treatment, respectively.

In the present series, intravitreal expansile gas injection, with or without intravitreal rtPA injection, was successful in achieving anatomic displacement of submacular blood. There has been some doubt regarding the role of rtPA in the management of SMH, in particular, whether intravitreal rtPA can access the subretinal space in sufficient quantities to induce clot liquefaction. There are several small clinical series to suggest that a gas bubble alone is enough to displace submacular blood and the role of rtPA remains uncertain. It has been shown that albumin, a protein comparable in molecular size to rtPA, can diffuse across the intact retina. Furthermore, tenecteplase, a synthetic variant of rtPA with almost identical molecular weight and size, has been shown to be able to penetrate through all layers of the retina following intravitreal injection in a porcine model.^28^ Further clinical and basic science investigations are required to elucidate the role of rtPA in this technique.

Expansile gas and rtPA physically shift the SMH away from the fovea and minimize scar formation. Anti-VEGF treatment can be given prior to, or simultaneous with pneumatic displacement of the submacular hemorrhage. Meyer et al^27^ used intravitreal bevacizumab simultaneously with intravitreal rtPA and SF6, but we used intravitreal bevacizumab after pneumatic displacement of the submacular hemorrhage. More studies are needed to identify the preferred timing of intravitreal bevacizumab in these cases.

In summary intravitreal expansile gas injection, with or without recombinant tissue plasminogen activator, followed by intravitreal bevacizumab injection seems to result in visual improvement in eyes with SMH secondary to neovascular AMD which may otherwise have a poor visual outcome.

## Figures and Tables

**Table 1:** Patient characteristics

Case	Age (years)	Duration of symptoms (days)	Treatment	Preoperative VA (logMAR)	Final VA (logMAR)
Case 1	80	3	SF6+rtPA+IVB	1	0.18
Case 2	83	3	SF6+IVB	1.3	0.7
Case 3	80	7	SF6+rtPA+IVB	1.5	0.7
Case 4	60	9	SF6+IVB	1.6	1
Case 5	75	10	SF6+IVB	1	0.3

VA, visual acuity; SF6, sulfur hexafluoride; rtPA, recombinant tissue plasminogen activator; IVB, intravitreal bevacizumab
